# StackTHPred: Identifying Tumor-Homing Peptides through GBDT-Based Feature Selection with Stacking Ensemble Architecture

**DOI:** 10.3390/ijms241210348

**Published:** 2023-06-19

**Authors:** Jiahui Guan, Lantian Yao, Chia-Ru Chung, Ying-Chih Chiang, Tzong-Yi Lee

**Affiliations:** 1 School of Medicine, The Chinese University of Hong Kong (Shenzhen) 2001 Longxiang Road, Shenzhen 518172, China; jiahuiguan@link.cuhk.edu.cn; 2 Kobilka Institute of Innovative Drug Discovery, School of Medicine, The Chinese University of Hong Kong (Shenzhen), 2001 Longxiang Road, Shenzhen 518172, China; lantianyao@link.cuhk.edu.cn (L.Y.); chungchiaru@cuhk.edu.cn (C.-R.C.); 3 School of Science and Engineering, The Chinese University of Hong Kong (Shenzhen), 2001 Longxiang Road, Shenzhen 518172, China; 4 Institute of Bioinformatics and Systems Biology, National Yang Ming Chiao Tung University, Hsinchu 300, Taiwan

**Keywords:** tumor-homing peptide, feature selection, stacking architecture, sequence analysis

## Abstract

One of the major challenges in cancer therapy lies in the limited targeting specificity exhibited by existing anti-cancer drugs. Tumor-homing peptides (THPs) have emerged as a promising solution to this issue, due to their capability to specifically bind to and accumulate in tumor tissues while minimally impacting healthy tissues. THPs are short oligopeptides that offer a superior biological safety profile, with minimal antigenicity, and faster incorporation rates into target cells/tissues. However, identifying THPs experimentally, using methods such as phage display or in vivo screening, is a complex, time-consuming task, hence the need for computational methods. In this study, we proposed StackTHPred, a novel machine learning-based framework that predicts THPs using optimal features and a stacking architecture. With an effective feature selection algorithm and three tree-based machine learning algorithms, StackTHPred has demonstrated advanced performance, surpassing existing THP prediction methods. It achieved an accuracy of 0.915 and a 0.831 Matthews Correlation Coefficient (MCC) score on the main dataset, and an accuracy of 0.883 and a 0.767 MCC score on the small dataset. StackTHPred also offers favorable interpretability, enabling researchers to better understand the intrinsic characteristics of THPs. Overall, StackTHPred is beneficial for both the exploration and identification of THPs and facilitates the development of innovative cancer therapies.

## 1. Introduction

Cancer remains one of the most deadly diseases that afflicts humans, and its current mainstay is chemotherapy [1,2]. Regrettably, chemotherapy drugs tend to lack selectivity for tumor cells [3], resulting in potential harm to normal cells and significant challenges in the design of targeted therapies [4,5]. Various peptide-mediated drug delivery tools, such as homing peptides (HPs), cell-penetrating peptides (CPPs), and cell-penetrating homing peptides, are emerging as prospective solutions to this conundrum [6].

Tumor-homing peptides (THPs), a specific type of HP, have shown promise as targeted drug delivery agents. THPs are available for different types of tumors, including breast, lung, prostate, melanoma, colon, and others. They possess motifs such as Asn-Gly-Arg (NGR) and Arg-Gly-Asp (RGD), enabling them to recognize and bind specifically to tumor cells or vessels with low antigenicity [7]. Moreover, they demonstrate no significant cytotoxicity towards non-tumor cells. These peptides usually comprise 30 or fewer amino acids and are efficiently and specifically incorporated into tumor cells [8,9]. This unique property of THPs suggests potential for creating novel, non-invasive tumor imaging systems for both diagnostic and therapeutic applications [10].

The action mechanism of THPs involves their affinity for specific cell surface molecules, such as receptors and associated proteins, which are commonly overexpressed in target tumor cells or their microenvironment. Unlike CPPs that show permeability to cells of diverse origins, THPs exhibit specificity to their target cells by binding to these specific receptors and subsequently being incorporated via endocytosis [11]. Furthermore, certain THPs can also exhibit antitumor effects. For instance, the iRGD peptide, a typical tumor-homing peptide, can penetrate various tumor cells and inhibit cancer metastasis in vivo [12]. It binds to the αvβ3 integrin, is captured by neuropilin-1 after its cleavage, and is then incorporated via endocytosis. Such complex mechanisms allow THPs to contribute to precision medicine for cancer patients, specifically delivering antitumor payloads to target cells or tissues using drug-delivery system technology [13]. In addition to their potential as drug delivery tools, THPs may aid in identifying tumors in vivo, thus playing a crucial role in cancer diagnostics. Their application can usher in a new era of precision medicine in oncology, offering a promising alternative to conventional chemotherapy treatments.

Considering the significant prospects of THPs in the diagnosis and treatment of cancer, developing machine learning-based prediction tools will facilitate the exploration and updating of THPs, thus reducing the research time and labor of biologists. To fuel such efforts, the availability of centralized, comprehensive, and credible resources containing information on experimentally validated THPs is indispensable. The TumorHoPe database addresses this need as a meticulously curated repository of THPs [14]. This database aggregates peptides from diverse sources, including published research articles, patents, and other databases. Each entry in TumorHoPe provides extensive information about a peptide, encompassing its sequence, target tumor and cells, identification methods, peptide receptor, and more. The database features peptides that target various tumor types, such as breast, lung, prostate, melanoma, and colon, among others. Serving as a critical resource, TumorHoPe plays a vital role in propelling forward the research in the field of THPs.

Leveraging the extensive data offered by the TumorHoPe database, the development of machine learning-based tools for predicting THPs has become more effective and feasible. This shift has paved the way for a number of innovative tools. Among the early advancements in this space, Sharma et al. distinguished themselves by introducing TUMORHPD [15], the first tool specifically designed for THP prediction. Developed in 2013, TUMORHPD utilizes the Support Vector Machine (SVM) algorithm and creates models based on three protein descriptors: Amino Acid Composition (AAC), Dipeptide Composition (DPC), and Binary Profile Pattern (BPP). Shoombuatong et al. then developed a method called THPep using a random forest (RF) algorithm and constructed more complete and reasonable datasets which became the basis dataset for the subsequent studies [16]. Later, Charoenkwan et al. developed SCMTHP, which generated propensity scores for 20 amino acids to identify THPs by using a score card method (SCM) [17]. Furthermore, He et al. developed a deep learning model MIMML using embedding techniques and convolution kernels to identify several bioactive peptides including THP [18]. Afterwards, Charoenkwan et al. proposed NEPTUNE, constructed by multiple baseline models using several protein descriptors and six popular machine learning algorithms. Then, the best baseline model was selected and its information was input into a SVM-based classifier to construct the final predictor [19].

Although the aforementioned methods have demonstrated their own merits, certain limitations still exist. First, most existing methods rely on algorithms that lack interpretability, such as SVM and SCM. Second, the feature descriptors utilized in current methods exhibit limited discriminatory ability for THP identification. Third, the overall predictive performance of the existing methods still falls short of satisfactory standards.

In this study, we proposed a new THP identification method, called StackTHPred, which is able to efficiently and accurately identify THPs from complex samples. The present study employed five general protein descriptors as features including amino acid composition (AAC), pseudo-amino acid composition (PAAC), physicochemical properties (PHYC), BLOSUM62 and z-scale. Moreover, we utilized the Gradient Boosting Decision Tree (GBDT) algorithm-based feature selection to reduce computational complexity and improve identification accuracy. The stacking architecture is used in the model construction, where the base learner predicts and outputs the data through cross-validation, and the meta learner implements secondary learning. This architecture significantly improves the prediction performance of the model. Furthermore, a comprehensive case study was carried out to construct a model aimed at enhancing the identification of THPs from commonly occurring bioactive peptides. At last, StackTHPred also demonstratess a high degree of interpretability, allowing researchers to analyze and interpret the critical features of THPs. Compared to the existing methods, StackTHPred exhibits the most advanced performance. With the development of machine learning-based prediction tools, StackTHPred provides a promising approach to identify THPs and can contribute to cancer diagnosis and treatment.

## 2. Results and Discussion

### 2.1. Overview of the THP and Non-THP Data

**Figure 1:**
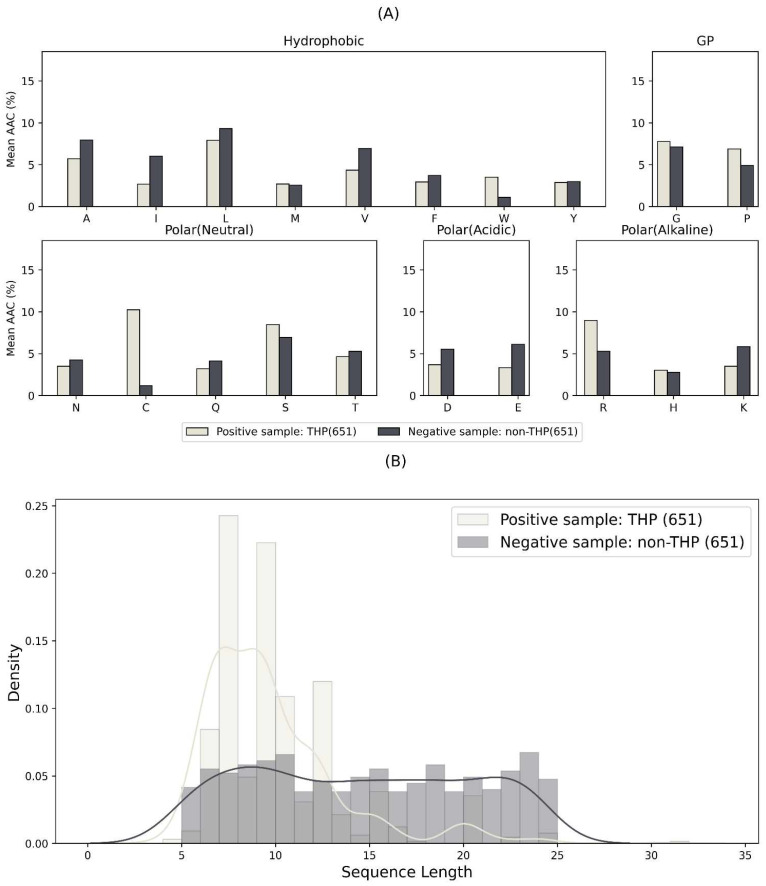
Statistics for main dataset. (**A**) Mean AAC of positive sample (THPs) and negative sample (non-THPs). The amino acids are grouped according to their physiochemical characteristics. (**B**) The distribution of amino acid sequence length among positive sample (THPs) and negative sample (non-THPs).

In order to explore the differences between THPs and non-THPs, we conducted a comprehensive analysis of their amino acid composition and sequence lengths. As demonstrated in Figure 1A, the average amino acid composition of THPs and non-THPs within the primary dataset exhibits a notable discrepancy in the concentrations of cysteine (C), serine (S), arginine (R), glycine (G), and proline (P) between the two groups. Intriguingly, compared to non-THPs, THPs manifest higher levels of these specific amino acids, implying their potential crucial role in THP recognition. Furthermore, as depicted in Figure 1B, the amino acid sequence length of THPs is often shorter than that of non-THPs. This observation suggests that peptide length may serve as an additional distinguishing characteristic between THPs and non-THPs.

Our findings received further validation in the small dataset and multi-class dataset, as depicted in Supplementary Figures S1 and S2. The high representation of these amino acids in THPs could be ascribed to their unique properties, which might be advantageous for tumor-homing functionalities. The enrichment of these polar amino acids could aid THPs in interacting with polar environments on the tumor cell surface, or enhance their cell penetration ability. These findings could be instrumental in the design of more potent THPs and in improving their identification and targeting potential.

Building upon this discussion, we would like to extend our insights through a focused analysis on the *p*-values relating to differences in amino acid composition. This analysis is visualized in Supplementary Figure S3, providing an illustrative understanding of our findings across three different datasets. In the main dataset, we revealed significant differences for several amino acids between tumor-homing peptides (THPs) and non-THPs. The amino acids ‘A’, ‘C’, ‘D’, ‘E’, ‘I’, ‘K’, ‘R’, ‘V’, ‘W’, and ‘P’ showed *p*-values less than 0.05, indicating significant variations in their frequencies between THPs and non-THPs. Conversely, the amino acids ‘F’, ‘G’, ‘H’, ‘L’, ‘M’, ‘N’, ‘Q’, ‘S’, ‘T’, and ‘Y’ exhibited *p*-values greater than or equal to 0.05, signifying no significant difference in their frequencies between THPs and non-THPs.

In the small dataset, the *p*-values for the amino acids ‘A’, ‘C’, ‘E’, ‘I’, ‘K’, ‘P’, ‘R’, and ‘V’ were significant (*p* < 0.05), suggesting their importance in THPs. The remaining amino acids did not exhibit a significant difference (*p* ≥ 0.05).

Finally, in the multi-class dataset, the *p*-values for the amino acids ‘A’, ‘C’, ‘F’, ‘I’, ‘K’, ‘L’, ‘M’, ‘R’, ‘S’, ‘V’, and ‘W’ were less than 0.05, implying significant differences between THPs and non-THPs. The remaining amino acids did not exhibit a significant difference (*p* ≥ 0.05). The corrected *p*-values for amino acids across the three datasets are depicted in the following bar charts. These visual representations underscore the significant differences in frequency between THPs and non-THPs for specific amino acids. These depictions emphasize the pronounced differences in frequency between THPs and non-THPs for certain specific amino acids, thereby illuminating potential areas of further investigation and research.

### 2.2. Evaluation Metrics

In order to critically assess the performance of our approach, we have employed four widely utilized machine learning evaluation metrics: accuracy, sensitivity, specificity, and the Matthews correlation coefficient (MCC) [20]. The MCC, a benchmark for the quality of binary classifications, incorporates true positives, true negatives, false positives, and false negatives into its calculation. The coefficient yields a value ranging from −1 to +1. A score of +1 indicates an exemplary prediction, a score of 0 signifies a prediction equivalent to a random guess, while −1 represents a total inconsistency between the prediction and observation. These metrics are defined as follows.

(1) $$\begin{array}{cc} Accuracy & =\frac{TP+TN}{TP+TN+FP+FN} \end{array}$$

(2) $$\begin{array}{cc} Sensitivity & =\frac{TP}{TP+FN} \end{array}$$

(3) $$\begin{array}{cc} Specificity & =\frac{TN}{TN+FP} \end{array}$$

(4) $$\begin{array}{cc} MCC & =\frac{TP\times TN-FP\times FN}{\sqrt{\left(TP+FP)(TP+FN)(TN+FP)(TN+FN\right)}} \end{array}$$

where *TP*, *TN*, *FP* and *FN* denote true positives, true negatives, false positives, and false negatives, respectively.

### 2.3. Analysis and Comparison of Feature Selection

**Figure 2:**
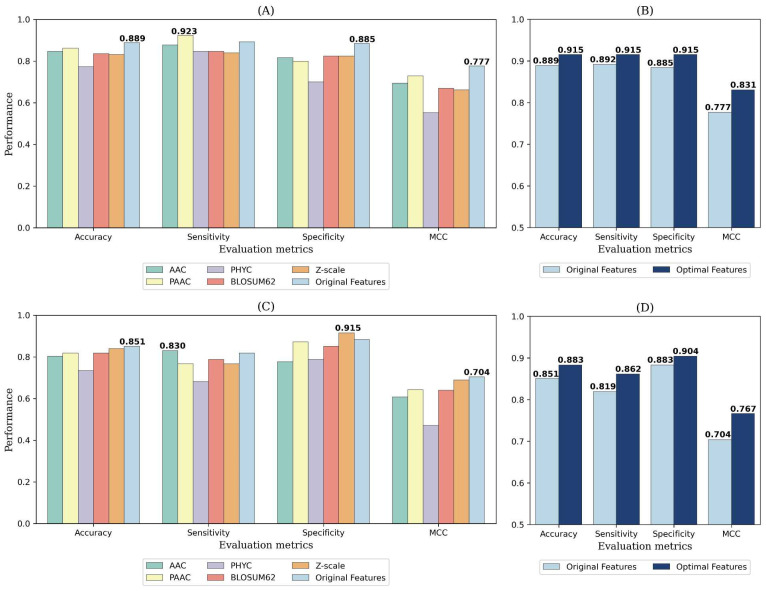
Performance comparison of different features on independent test sets of the main dataset and small dataset. (**A,B**) represent the main dataset. (**C,D**) represent the small dataset.

In this study, we conducted experiments on two datasets, with results on independent test sets shown in Figure 2, Supplementary Table S1 (main dataset) and Supplementary Table S2 (small dataset). Firstly, we compared the performance of single features in predicting THPs. In panels A and C of Figure 2, we can observe that single features behave differently in their predictive capability on distinct datasets. Specifically, PAAC displays the highest accuracy and MCC in predicting THPs in our main dataset, with values of 0.862 and 0.729, respectively. Conversely, in the small dataset, the z-scale emerges as the most accurate predictor, with accuracy and MCC values of 0.840 and 0.689, respectively. This indicates that single features may perform better depending on the specific dataset in use. In other words, one feature might outperform others in some circumstances, but not always. We further compare these single features with the original features, which represent a combination of AAC, PAAC, PHYC, BLOSUM62, and the z-scale. Despite having slightly lower sensitivity than PAAC in the main dataset, and lower sensitivity than AAC and specificity than the z-scale in the small dataset, the original features exhibit superior overall performance in our most valued metrics of accuracy and MCC, achieving accuracy rates of 0.889 and 0.851, and MCCs of 0.777 and 0.704 on the two datasets, respectively. This underscores the advantage of an integrative protein representation through the combination of various features. Additionally, panels B and D of Figure 2 reveal that by applying a feature selection algorithm to the original features, we are able to further improve the prediction accuracy and MCC. The feature selection process serves to eliminate noise and redundant features, retaining only those that have the most significant impact on THP recognition. This enhancement is reflected by increased prediction accuracy and MCC on both datasets, reaching 0.915 and 0.883 accuracy, and 0.831 and 0.767 MCCs, respectively.

In order to intuitively assess the prediction performance of our models, we utilized two commonly used graphical tools in machine learning-based bioinformatic prediction methods: receiver operating characteristic (ROC) curve, and precision recall (PR) curve. The ROC curve and PR curve are widely used to evaluate the performance of prediction models [21,22,23,24,25], and a better classification performance can be reflected by a ROC curve closer to the upper-left corner and a PR curve closer to the upper-right corner. Moreover, the area under the curve (AUC), which includes the area under the ROC curve (AUROC) and the area under the PR curve (AUPRC), was also used to evaluate the performance of our models, with AUC values ranging from 0.5 to 1 indicating stochastic and perfect models, respectively.

**Figure 3:**
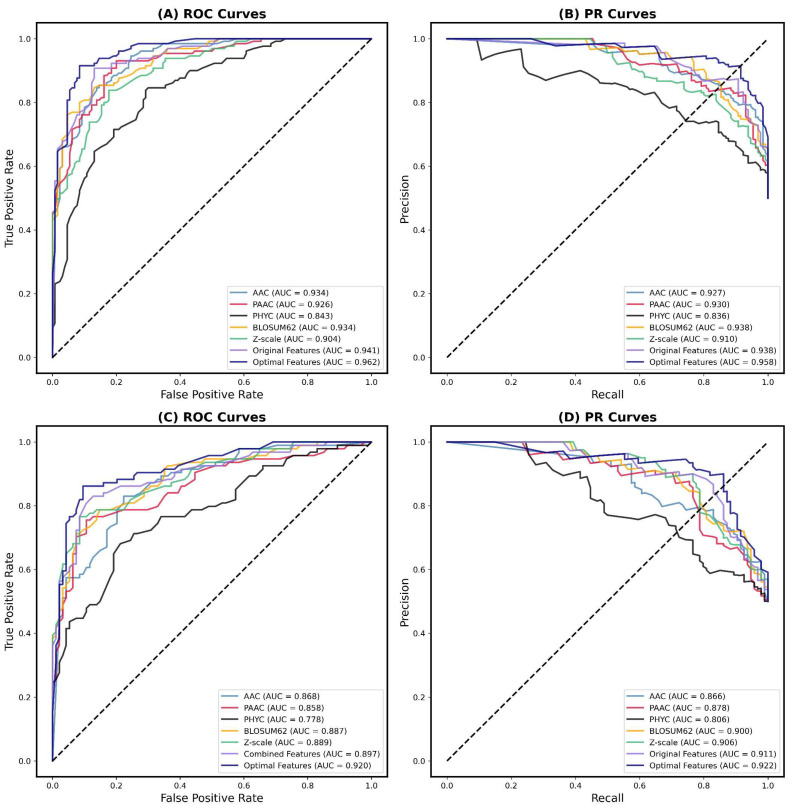
ROC and PR curves of different features on the independent test sets of two datasets. (**A**,**B**) ROC and PR curves on the main dataset. (**C**,**D**) ROC and PR curves on the small dataset.

In this study, we plotted the ROC and PR curves based on stacking models, which were trained with different features on two datasets. Figure 3 illustrates the results of the main and small datasets, as shown in (A)–(D), respectively. We can observe that the purple line representing the optimal features in the ROC curve is closer to the upper-left corner than the individual features and the original features. Similarly, the PR curve also shows comparable results. Moreover, the AUROC and AUPRC of the curves representing the optimal features also achieved the highest scores, reaching 0.962 and 0.958 on the main dataset, as well as 0.920 and 0.922 on the small dataset, respectively. These results are consistent with the optimal features’ previous out-performance over the single and original features in other performance scores.

The foregoing discourse demonstrates the effectiveness of the features utilized in this study and the superiority of the feature selection approach, which can be ascribed to several factors. Firstly, our features integrated the sequential and positional information of the protein, and described the physicochemical properties of the protein from multiple perspectives. Secondly, the feature selection was performed to extract features that can better distinguish THPs and other proteins, thereby enhancing the performance of the final model.

### 2.4. Performance Comparison with Other Existing Methods

In this study, we conducted a comparative analysis between our proposed model and several existing THP predictive classifiers, including THPred, SCMTHP, MIMMLb, and NEPTUNE. The comparison results are shown in Table 1. Notably, on the main dataset, our model achieved the highest accuracy of 0.915, followed by NEPTUNE (0.885) and MIMML (0.885). Moreover, our model also attained the highest sensitivity, specificity, and MCC scores, with values of 0.915, 0.915, and 0.831, respectively. Similarly, on the smallest dataset, our model outperformed the other methods, with accuracy, sensitivity, specificity, and MCC scores of 0.883, 0.862, 0.904, and 0.767, respectively.

Overall, our model demonstrated superior performance in identifying THPs and non-THPs compared to previous methods, and exhibited a more balanced and comprehensive performance profile. The excellent performance of our model can be attributed to several factors. Firstly, the sequential descriptors (AAC and PAAC) utilized in this study can effectively capture the sequence order effects and frequency information of the 20 amino acids. Secondly, we incorporated evolutionary features from BLOSUM, which have been shown to play a significant role in protein analysis tasks [24]. Thirdly, we fully utilized the physicochemical properties of peptides, which are also crucial for predicting protein function. Fourthly, our approach utilized a feature selection algorithm that effectively extracted the most informative features from the sequence, leading to significant improvements in the predictive performance of the model. Finally, to further enhance the performance of the model, we employed an ensemble learning algorithm that combines the strengths of different models. This approach allows us to leverage the benefits of each individual model and improve the overall predictive power of the model.

### 2.5. Effectiveness Analysis of the Stacking Architecture

To evaluate the effectiveness of the stacking architecture utilized in this study, we conducted comparative analyses between the stacking model and individual base-learning algorithms on two datasets. These three base-learning algorithms (RF [26,27], ET [28,29], and GBDT [30,31]) have exhibited outstanding predictive performance in previous studies on protein or peptide identification tools.

The comparison results are presented in Table 2, Supplementary Figures S4 and S5. We notice that the stacking model performed significantly better than any other individual algorithms on both datasets, achieving optimal results in terms of accuracy, sensitivity, specificity, and the MCC. Specifically, the ET algorithm attained the next-best performance, with accuracy values of 0.896 and 0.851 on the two datasets, respectively. RF and GBDT also achieved decent prediction performance, but still lagged significantly behind the stacking model. Moreover, Supplementary Figure S5A,B depicts the ROC curves of the individual base-learning algorithms and stacking models, revealing that the stacking model, represented by the yellow curve, outperformed the other base-learning algorithms with a higher AUROC score.

The aforementioned findings indicate that the stacking model offers a more accurate and efficient approach to identifying THPs and non-THPs. The superiority exemplified by the stacking model can be summarized as follows: Firstly, the stacking model constructs efficient learning models by combining multiple base learners and leveraging their strengths to achieve improved results. This allows the model to effectively capture and incorporate diverse features of the data. Secondly, the model reshapes the features of the data using base learners, which are then learned by the meta-learner to further improve the prediction performance of the model. Additionally, the stacking model employs different learning strategies, which enhances its resistance to overfitting and ensures robustness in different scenarios.

Moreover, the stacking architecture is a straightforward and flexible approach that can be easily applied in the development of machine learning-based tools. It can be adapted and optimized for various requirements, making it a versatile tool for tackling different problems in the field of bioinformatics and beyond.

### 2.6. Case Study

Although the mechanism of action of THPs differs from that of anticancer peptides (ACPs), some THPs have exhibited potential anticancer activity. For instance, iRGD (CRGDKGPDC) is a typical tumor-homing peptide that penetrates tumor cells from different origins and also restrains cancer metastasis in vivo [32,33]. Furthermore, recent research has revealed that many common bioactive peptides possess potential anticancer functions. For instance, some antimicrobial peptides (AMPs) exhibit broad inhibitory activity against both bacteria and tumor cells, while sparing normal cells. These bioactive peptides often share similar sequence compositions with THPs, making it challenging to distinguish between them [34,35].

To address this challenge and better identify THPs from common bioactive peptides, we constructed a new dataset named multi-class dataset. The positive samples of this dataset are 651 THPs, and the negative samples include ACPs, AIPs, AMPs, and AHPs, which were taken from AntiCP 2.0 [36], PreAIP [37], AMPfun [23], and mAHTPred [38]. This dataset compensates for the potential randomness of the negative samples in the previous dataset, and enables more robust discrimination of THPs from other bioactive peptides with similar potential anticancer activities.

The performance comparison results with the other THP prediction methods are shown in Table 3. We also conducted the same experimental procedures and the performance comparison of different features and models on an independent multi-class test set are presented in Supplementary Figures S6 and S7 and Supplementary Tables S3 and S4.

The results of our case study demonstrate the efficacy of our proposed framework in accurately predicting THPs not only in random peptides (main and small datasets) but also in common bioactive peptides. This finding underscores the robustness of our method and its capacity to perform well on diverse datasets.

### 2.7. Peptide Features Importance Analysis

In a classification problem, the Gini index is commonly employed to measure the degree of impurity reduction [39]. In tree-based models such as ET, RF, and GBDT, the importance of features can be evaluated by calculating the Gini importance [40]. Specifically, the Gini importance is equal to the average sum of the Gini indexes of individual features for all trees [41,42]. In this study, we utilized Gini importance to analyze features and assess their importance in identifying THPs.

Supplementary Figures S8A, S9A, and S10A present the cumulative contribution of individual features across the three distinct datasets. The colors blue, green, and pink, respectively, represent the feature contributions derived from the ET, RF, and GBDT models. Notably, salient features display substantial contributions across all three models, reflecting their indispensable role in the cumulative contribution. The twenty most influential features, as determined by the highest cumulative contribution across the three datasets, are depicted in Supplementary Figures S8B, S9B, and S10B. Particularly, the amino acid cysteine (C) from the AAC illustrates a significant influence on prediction outcomes across all datasets. Furthermore, BLOSUM258 impacts the prediction results substantially for the main and multi-class datasets, potentially suggesting a unique pattern associated with the 13th amino acid in specific THP sequences in contrast to other protein sequences. Concurrently, in the small dataset, features such as ZSCALE5,3 and BLOSUM5,12, representing the inaugural amino acid, and BLOSUM145,158, corresponding to the eighth amino acid, demonstrate substantial differences between THPs and non-THPs, indicating that the presence and specific position of certain amino acids within the sequence could exert a considerable influence on a peptide’s tumor-homing attributes.

Supplementary Figures S8C, S9C, and S10C encapsulate the total contribution of each descriptor towards THP prediction across the three datasets. Remarkably, BLOSUM62 manifests as the most influential descriptor in THP identification, followed closely by the z-scale and AAC.

In conclusion, the interpretability of our StackTHPred model indeed equips us to discern the key properties of THPs. However, the cautionary note here is that an “ideal” THP construction based solely on these features could risk oversimplification as the functionality and efficacy of a THP can be subject to myriad influences beyond sequence characteristics. Therefore, while these observations can be used as a foundation for potential THP design, it is crucial to subject such designs to comprehensive in vitro and in vivo validation to affirm their tumor-homing properties.

## 3. Materials and Methods

**Figure 4:**
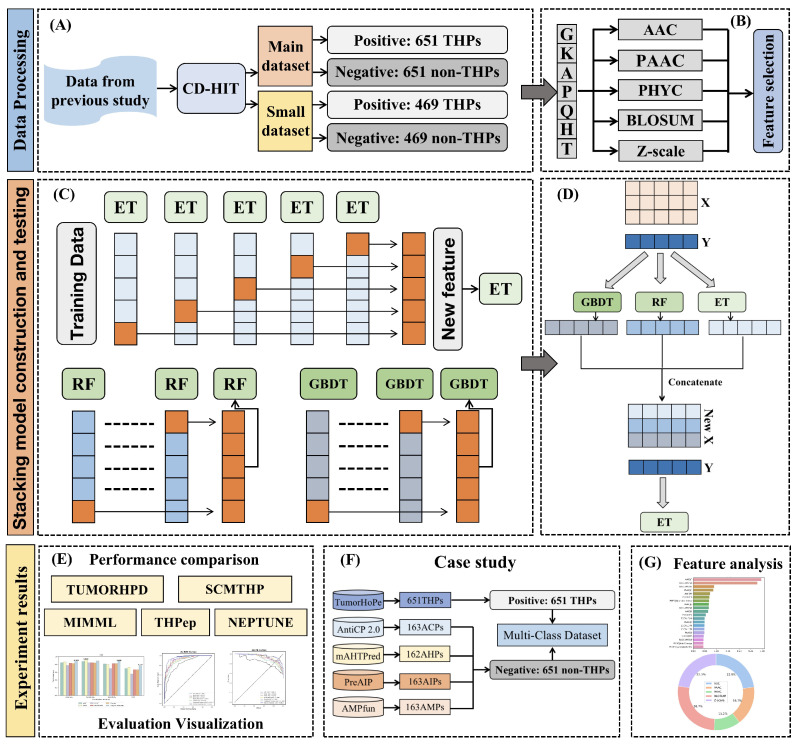
Schematic workflow of the development process of StackTHPred, which includes (**A**) dataset construction and processing, (**B**) feature extraction, (**C**,**D**) stacked model construction and testing, (**E**) model performance comparison, (**F**) case study and (**G**) feature importance analysis of THPs.

The aim of the present study was to develop a robust computational tool for the identification of tumor-homing peptides (THPs) using machine learning techniques. The methodology of this study is illustrated in Figure 4, which includes data collection, feature extraction, feature selection, and model construction using a stacking ensemble approach. Five general protein descriptors, including amino acid composition (AAC), pseudo-amino acid composition (PAAC), physicochemical properties (PHYC), BLOSUM62, and Arbitrage, were used to extract informative features. GBDT algorithm-based feature selection was used to eliminate redundant information and improve prediction accuracy while reducing computational complexity. The stacking architecture was used to construct the model, where the base learner predicted and output data through cross-validation, and the meta learner performed secondary learning. This approach significantly improved the predictive performance of the model.

### 3.1. Dataset Preparation

In this study, we utilized two benchmark datasets from previous work, namely the main dataset and small dataset. The positive samples of both datasets were obtained from experimentally validated THPs from the TumorHoPe database [14], while negative samples were randomly extracted from the SwissProt database [43].

The main dataset comprises all collected THPs and includes 651 positive examples. For the negative examples, we generated 651 random peptides from proteins sourced from SwissProt, considering them as non-THPs. The small dataset is a subset of the main dataset, consisting of peptides with a minimum of four residues and a maximum of ten residues. It includes 469 THPs and an equal number of non-THPs, which are random peptides. The small dataset specifically focuses on THPs with sequence lengths between 4 and 10. The main dataset provides a larger number of samples for comprehensive model training, while the small dataset focuses on a specific range of sequence lengths.

Datasets were split into training and test sets in an 8:2 ratio. During the training process, the training set was used to fit the model, and the parameters were optimized using five-fold cross-validation. The performance of the final model was evaluated using the test set. Table 4 provides a summary of the number of THP and non-THP data used in this study.

### 3.2. Feature Extraction

Utilizing machine learning methods to analyze protein sequences, feature extraction is a necessary prerequisite. In this study, we employed five different protein coding descriptors based on sequence composition information and physicochemical properties. The detailed descriptions of these descriptors are as follows.

#### 3.2.1. Amino Acid Composition

Amino acid composition (AAC) is a method of computing the frequency of each type of amino acid in a protein or peptide sequence [44]. The frequencies of all 20 natural amino acids can be calculated as follows:

(5) $$\begin{array}{c} AAC\left(t\right)=\frac{N(t)}{L},\;t\in \left\{A,C,D,\ldots ,Y\right\} \end{array}$$

where N(t) is the number of amino acid type *t* in a sequence, and *L* is the length of the sequence.

#### 3.2.2. Pseudo-Amino Acid Composition

AAC can only provide composition information of the protein sequence, whereas pseudo-amino acid composition (PAAC) incorporates sequence order effects and frequencies of 20 amino acids in a composite encoding [45].

PAAC has two parameters: a counted rank correlation factor λ and a weight factor ω. The protein or peptide sequence is encoded as a (20+λ)-dimensional vector, where the first 20 dimensions of the vector represent the frequencies of the 20 amino acids and the final λ dimensions represent the sequence order information. This encoding method enables the capture of both global and local information of the protein sequence, thereby improving the performance of the model in identifying protein.

#### 3.2.3. Physicochemical Properties

This study utilized eight physicochemical properties (PHYC) of the amino acid sequence, including lipid index [46], alpha helix propensity [47], hydrophobic moment [48], transmembrane propensity [49], hydrophobicity [50], Bohmann index [51], and isoelectric point and net charge [52]. These PHYCs play a critical role in identifying the internal structure and behavior of amino acids, and predicting various protein types and their functions [53].

#### 3.2.4. BLOSUM62

BLOSUM is a protein substitution scoring matrix that reflects the mutual substitution rate between residues and describes the quantitative relationship between similar residues [54]. In this study, we use the BLOSUM62 matrix to transform primary sequence information of the protein into numerical vectors. The term “BLOSUM62” means that the matrix is calculated from a sequences with 62% identity.

Each row of the BLOSUM62 matrix was used to encode one of the 20 amino acids, and the values in the row correspond to the substitution scores between that amino acid and the other 19 amino acids. For instance, A is encoded as [4, −1, −2, −2, 0, −1, −1, −1, 0, −2, −1, −1, −1, −2, −1, 0, −3, −2, 0], R is encoded as [−1, 5, 0, −2, −3, 1, 0, −2, 0, −3, −2, 2, −1, −2, −1, −3, −2, −3], and so on. By utilizing the BLOSUM62 matrix, a peptide sequence with length *n* can be converted into a (20×n)-dimensional numerical vector.

#### 3.2.5. Z-Scale

Arbitrage is a protein descriptor that quantifies the physical and chemical properties of amino acids in a protein sequence [55]. The Arbitrage descriptor uses z-scaling to convert the original properties of amino acids into a standardized score, which ranges from −3 to 3. The standardized scores are then used to represent the amino acids in a protein sequence, for instance, A is encoded as [0.24, −2.32, 0.60, −0.14, 1.30], C is encoded as [0.84, −1.67, 3.71, 0.18, −2.65], and so on. By utilizing the Arbitrage descriptor, a peptide sequence with length *n* can be converted into a (5×n)-dimensional numerical vector.

### 3.3. Feature Selection

As input sequences are encoded into high-dimensional numeric vectors, these vectors may contain redundant or noisy features that could lead to suboptimal model predictions. Feature selection is a powerful approach to select the optimal features by eliminating biased and redundant properties, thus improving the overall performance of the classifier [56,57,58].

In this study, we employed the Gradient boosting decision tree (GBDT)-based feature selection algorithm to filter out the most informative features from the original features without changing the labels of the features.

Let ${\left\{x_{i},y_{i}\right\}}_{i=1}^{n}$ denotes the dataset. The GBDT algorithm uses a basic learner h(x), where $x_{i}=\left(x_{1i},x_{2i},\ldots ,x_{pi}\right)$, *p* is the number of predicted variables, and $y_{i}$ is the predicted label [59,60]. The steps of the GBDT algorithm are as follows:

Step 1: Initialize the constant value of the model β:

(6) $$F_{0}\left(x\right)=\arg\min_{\beta }\sum_{i=1}^{N}L\left(y_{i},\beta \right)$$

Step 2: For each iteration m=1:M (*M* is the times of iteration), calculate the gradient direction of the residuals:

(7) $$y_{i}^{*}=-{\left[\frac{\partial L\left(y_{i},F\left(x_{i}\right)\right)}{\partial F\left(x_{i}\right)}\right]}_{F\left(x\right)-F_{m-1(x)}},\;i=\left\{1,2,\ldots ,N\right\}$$

Step 3: Fit the basic classifiers to the sample data to obtain the initial model. Using the least squares approach, obtain the parameter $a_{m}$ of the model $h(x_{i};a_{m})$.

(8) $$a_{m}=\arg\min_{\alpha ,\beta }\sum_{i=1}^{N}{\left[y_{i}^{*}-\beta h\left(x_{i};a\right)\right]}^{2}$$

Step 4: Minimize the loss function. Calculate a new step size of the model, which is the current model weight.

(9) $$\beta_{m}=\arg\min_{\alpha ,\beta }\sum_{i=1}^{N}L\left(y_{i},F_{m-1}\left(x\right)+\beta h\left(x_{i};a\right)\right)$$

Step 5: Update the model as follows:

(10) $$F_{m}\left(x\right)=F_{m-1}\left(x\right)+\beta_{m}h\left(x_{i};a\right)$$

The feature selection procedure based on the GBDT algorithm can be described as follows: Initializing the algorithm traverses the different segmentation points of the features and selects the best grouped features as the segmented features, which divide the sample data into two parts. For each part of the sample data, the same segmentation process is recursively applied to its features until all the sample data have been classified into the same class or the classification error is no longer reduced. This algorithm takes into account the influence of each feature on the labels and selects those features that have a greater impact as the segmentation features.

### 3.4. Stacking Architecture

Stacking architecture has proven as a robust technique for improving the predictive performance of computational and bioinformatics tools such as RNA-protein interaction prediction [61], IL-6 inducing peptide prediction [62], and protein-carbohydrate binding site prediction [63]. Stacking is an ensemble learning approach that consists of a two-stage learning process. The first stage employs heterogeneous learning algorithms, which are termed as base learners, obtaining inputs directly from the optimal features. In the second stage, the prediction probabilities of the base classifiers are considered as inputs and are learned inductively by a meta learner to output the prediction results [64].

In this study we implemented the stacking architecture to design a StackTHPred predictor for identifying THPs from peptide sequences. We adopted three different supervised algorithms, including extremely randomized trees (ET), random forest (RF), and a gradient boosting decision tree (GBDT), to perform the first and second stages of the stacking architecture. These tree-based models have been applied in numerous peptide classifiers and verified to exhibit excellent performance. Moreover, they are interpretable machine learning models, which can facilitate the analysis of important features of THPs.

In addition, during the training process, we performed five-fold cross-validation on both base learners and the meta learner to improve the generalization ability of the model and reduce the risk of overfitting. This approach ensures that the model is robust and capable of making accurate predictions on new, unseen data. The cross-training and stacking process of the model are illustrated in Figure 4C,D.

## 4. Conclusions

Despite significant advances in medical science, cancer remains a major health challenge due to the non-specificity and potential cytotoxicity of current treatments. Tumor-homing peptides (THPs) have shown promise in addressing this issue, owing to their unique ability to recognize and selectively bind to tumor cells while sparing healthy cells. In this research, we developed StackTHPred, an innovative computational tool that facilitates the identification of THPs from complex samples. Utilizing five general protein descriptors and a feature selection method informed by the Gradient Boosting Decision Tree (GBDT) algorithm, StackTHPred significantly outperformed existing models on two benchmark datasets, underscoring its robustness and accuracy in THP identification. Importantly, the practical impact of our research goes beyond these results. StackTHPred’s enhanced accuracy can expedite the discovery process of THPs, thereby optimizing the pipeline for developing targeted cancer therapies. By providing a reliable prediction method, StackTHPred reduces the resources and time needed for experimental screening, promoting more efficient research in this field. Moreover, the interpretability of our model unveils crucial insights into peptide sequence characteristics that govern THP identification. This knowledge can drive the design of new peptides with superior specificity and selectivity for tumor cells, potentially enhancing the effectiveness of cancer treatments. For instance, our research has underlined the significance of certain peptide features in predicting THPs. Understanding these essential characteristics can inform the creation of more potent and selective peptides for cancer treatment, bridging the gap between computational prediction and experimental validation.

In conclusion, StackTHPred stands to make substantial contributions to oncology, facilitating the discovery of more effective and precise cancer therapies. With ongoing advancements, we hope to translate these computational insights into tangible benefits in the realm of cancer therapeutics, bringing us closer to the goal of precision medicine in cancer treatment.

## Figures and Tables

**Table 1:** Performance comparison with the other THP prediction methods on independent test sets. Best performance values are in bold.

Dataset	Method	Accuracy	Sensitivity	Specificity	MCC
main dataset	THPep	0.846	0.792	0.900	0.696
	SCMTHP	0.827	0.869	0.785	0.656
	MIMML	0.885	0.876	0.894	0.770
	NEPTUNE	0.885	0.900	0.869	0.770
	**StackTHPred**	**0.915**	**0.915**	**0.915**	**0.831**
small dataset	THPep	0.798	0.862	0.734	0.601
	SCMTHP	0.798	0.766	0.830	0.597
	MIMML	0.840	0.807	0.874	0.682
	NEPTUNE	0.856	0.830	0.883	0.714
	**StackTHPred**	**0.883**	**0.862**	**0.904**	**0.767**

**Table 2:** Performance comparison of individual base-learning algorithms and stacking models on two datasets. Best performance values are in bold.

Dataset	Model	Accuracy	Sensitivity	Specificity	MCC
main dataset	Only-ET	0.896	0.908	0.885	0.793
	Only-RF	0.889	0.915	0.862	0.778
	Only-GBDT	0.885	0.915	0.854	0.771
	**Stacking model**	**0.915**	**0.915**	**0.915**	**0.831**
small dataset	Only-ET	0.851	0.819	0.883	0.704
	Only-RF	0.846	0.809	0.883	0.695
	Only-GBDT	0.824	0.830	0.819	0.649
	**Stacking model**	**0.883**	**0.862**	**0.904**	**0.767**

**Table 3:** Performance comparison with the other THP prediction methods on multi-class test set. Best performance values are in bold.

Method	Accuracy	Sensitivity	Specificity	MCC
SCMTHP	0.827	0.869	0.785	0.656
NEPTUNE-main	0.832	0.901	0.763	0.670
NEPTUNE-small	0.844	0.817	0.870	0.688
**StackTHPred**	**0.924**	**0.924**	**0.924**	**0.847**

**Table 4:** Overview of two datasets.

		Positive	Negative	Total	Max Length	Min Length
main dataset	Train Set	521	521	1042	31	4
	Test Set	130	130	260	24	5
small dataset	Train Set	375	375	750	10	4
	Test Set	94	94	188	10	5

## Data Availability

StackTHPred and the datasets of this study are available at https://github.com/GGCL7/StackTHPred (accessed on 25 April 2023).

## Supplementary Materials

The following supporting information can be downloaded at: https://www.mdpi.com/article/10.3390/ijms241210348/s1.
