# Supplementary material for: StackTHPred: Identifying Tumor-Homing Peptides through GBDT-Based Feature Selection with Stacking Ensemble Architecture
Source: Int J Mol Sci. 2023 Jun 19;24(12):10348. doi: 10.3390/ijms241210348 (PMC10298859; doi:10.3390/ijms241210348)
Supplement: Supplementary file 1 [file ijms-24-10348-s001.zip › ijms-2391241-supplementary.pdf]

SUPPLEMENTARY MATERIALS

Table S1. Comparison of the performance of different features on main independent dataset.

Table S2. Comparison of the performance of different features on small independent dataset.

Table S3. Comparison of the performance of different features on multi-class independent dataset.

Table S4. Performance comparison of Individual and Stacking models on multi-class independent dataset.

Figure S1. Statistics for small dataset. (A) Mean AAC of positive sample (THPs) and negative sample (non-THPs). The amino acids are grouped according to their physiochemical characteristics. (B) The distribution of amino acid sequence length among positive sample (THPs) and negative sample (non-THPs).

Figure S2. Statistics for multi-class dataset. (A) Mean AAC of positive sample (THPs) and negative sample (non-THPs). The amino acids are grouped according to their physiochemical characteristics. (B) The distribution of amino acid sequence length among positive sample (THPs) and negative sample (non-THPs).

Figure S3. Corrected P-values for Amino Acid Composition Differences across Datasets. The bar charts depict the statistical significance of amino acid composition differences between THPs and non-THPs for (A) main dataset, (B) small dataset, and (C) multi-class dataset. The Y-axis denotes the corrected p-values, and the X-axis represents the individual amino acids. A lower p-value indicates a more significant difference in the frequency of a specific amino acid between THPs and non-THPs.

Figure S4. Performance comparison of individual base-learning algorithms and stacking models on two datasets. (A) represent main dataset. (B) represent small dataset.

Figure S5. Comparison of the ROC curves of of Individual and Stacking models on two datasets. (A)represents main dataset. (B)represents small dataset.

Figure S6. Performance comparison of different features on independent test sets of multi-class independent dataset.

Figure S7. ROC and PR curves of different features on the independent test set of multi-class independent dataset.

Figure S8. Features importance analysis on main dataset. (A) Visualize the cumulative

contribution of each feature, where blue, green and pink represent the feature contribution from ET, RF and GBDT, respectively. (B) Demonstrate the top twenty features with the highest cumulative contribution of each feature. (C) Illustrate the total contribution of each descriptor.

Figure S9. Features importance analysis on small dataset. (A) Visualize the cumulative contribution of each feature, where blue, green and pink represent the feature contribution from ET, RF and GBDT, respectively. (B) Demonstrate the top twenty features with the highest cumulative contribution of each feature. (C) Illustrate the total contribution of each descriptor.

Figure S10. Features importance analysis on multi-class dataset. (A) Visualize the cumulative contribution of each feature, where blue, green and pink represent the feature contribution from ET, RF and GBDT, respectively. (B) Demonstrate the top twenty features with the highest cumulative contribution of each feature. (C) Illustrate the total contribution of each descriptor.

Table S1. Comparison of the performance of different features on main independent dataset.

Feature	Accuracy	Sensitivity	Specificity	MCC
AAC	0.846	0.877	0.815	0.694
PAAC	0.862	0.923	0.800	0.729
PHYC	0.773	0.846	0.700	0.552
BLOSUM	0.835	0.846	0.823	0.669
Z-scale	0.831	0.839	0.823	0.667
Original Features	0.889	0.892	0.885	0.777
**Optimal Features**	**0.915**	**0.915**	**0.915**	**0.831**

Table S2. Comparison of the performance of different features on small independent dataset.

Feature	Accuracy	Sensitivity	Specificity	MCC
AAC	0.803	0.830	0.777	0.607
PAAC	0.819	0.766	0.872	0.642
PHYC	0.734	0.681	0.787	0.471
BLOSUM	0.819	0.787	0.851	0.640
Z-scale	0.840	0.766	**0.915**	0.689
Original Features	0.851	0.819	0.883	0.704
**Optimal Features**	**0.883**	**0.862**	0.904	**0.767**

Table S3. Comparison of the performance of different features on multi-class independent dataset.

Feature	Accuracy	Sensitivity	Specificity	MCC
AAC	0.832	0.832	0.832	0.664
PAAC	0.844	0.863	0.824	0.688
PHYC	0.802	0.847	0.756	0.606
BLOSUM62	0.878	0.886	0.87	0.756
Z-scale	0.882	0.87	0.893	0.764
Original Features	0.893	0.886	0.901	0.786
**Optimal features**	**0.924**	**0.924**	**0.924**	**0.847**

Table S4. Performance comparison of Individual and Stacking models on multi-class independent dataset.

Model	Accuracy	Sensitivity	Specificity	MCC
Only-ET	0.908	0.908	0.908	0.817
Only-RF	0.893	0.893	0.893	0.786
Only-GBDT	0.893	0.901	0.885	0.786
**Stacking model**	**0.924**	**0.924**	**0.924**	**0.847**

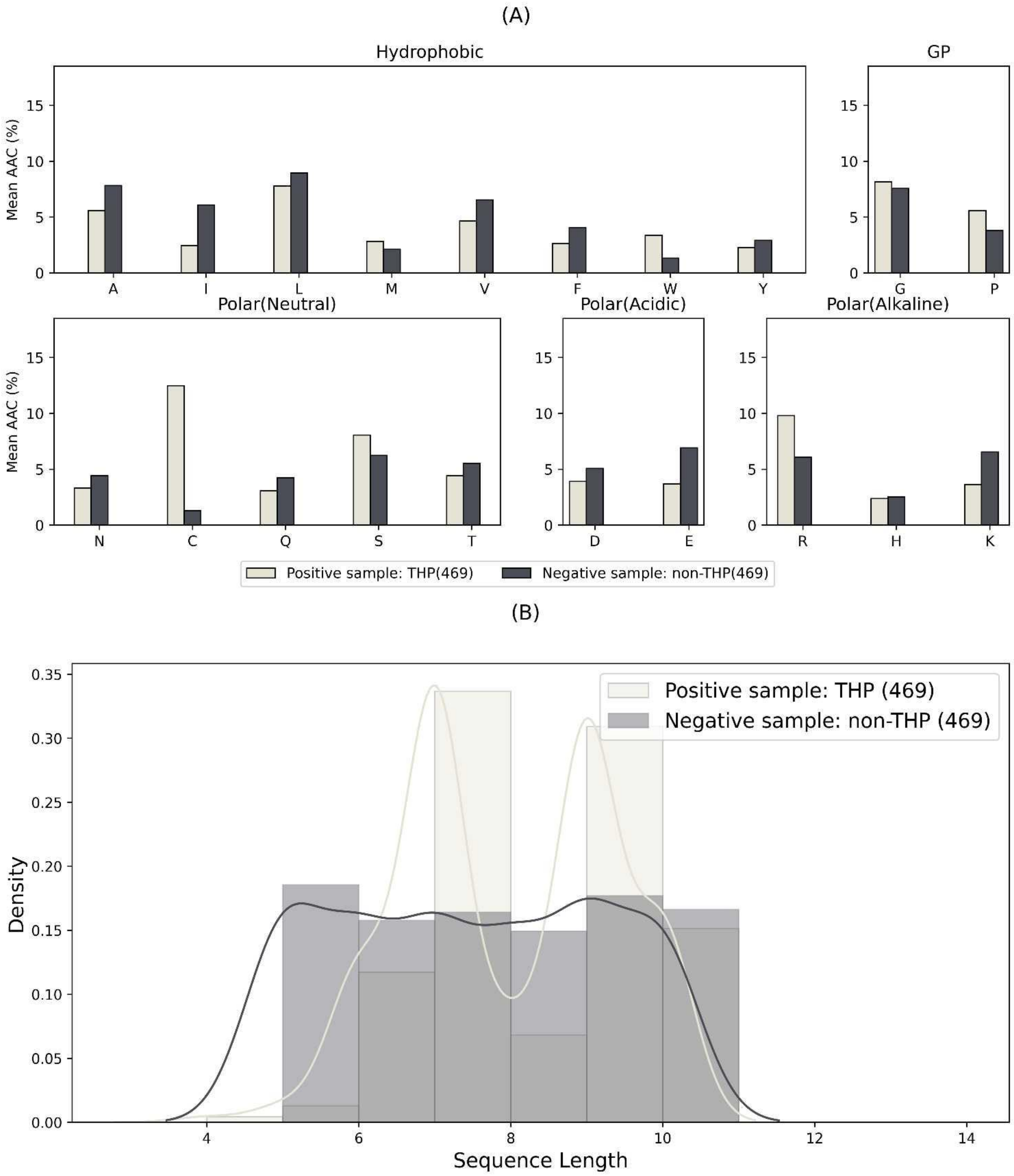

Figure S1. Statistics for small dataset. (A) Mean AAC of positive sample (THPs) and negative sample (non-THPs). The amino acids are grouped according to their physiochemical characteristics. (B) The distribution of amino acid sequence length among positive sample (THPs) and negative sample (non-THPs).

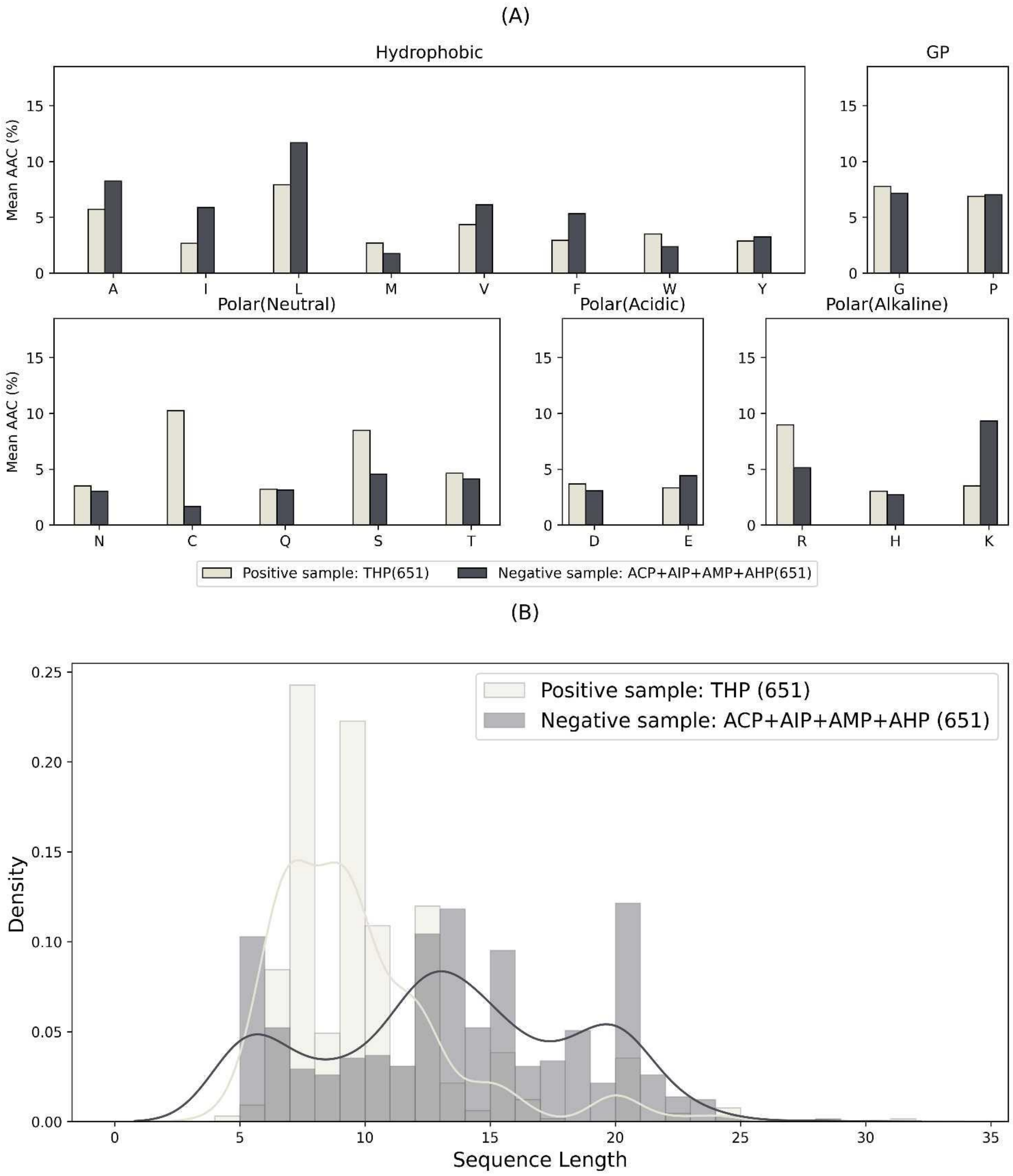

Figure S2. Statistics for multi-class dataset. (A) Mean AAC of positive sample (THPs) and negative sample (non-THPs). The amino acids are grouped according to their physiochemical characteristics. (B) The distribution of amino acid sequence length among positive sample (THPs) and negative sample (non-THPs).

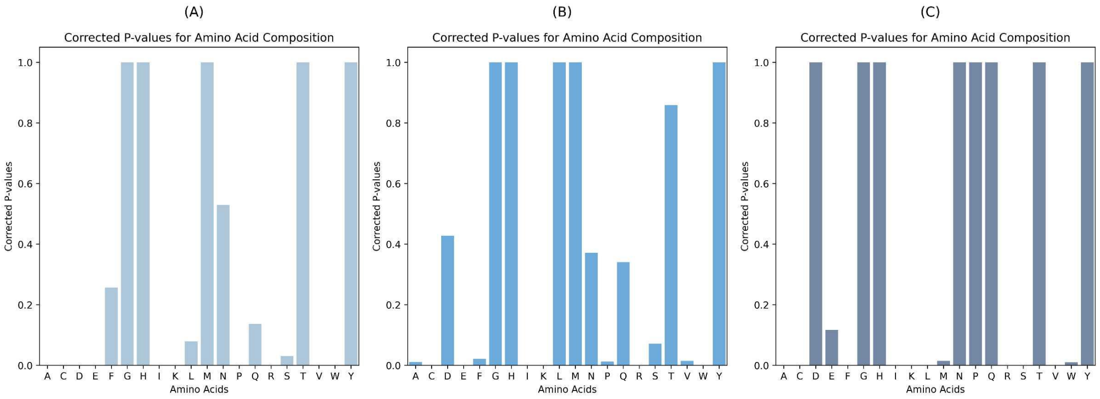

Figure S3. Corrected P-values for Amino Acid Composition Differences across Datasets. The bar charts depict the statistical significance of amino acid composition differences between THPs and non-THPs for (A) main dataset, (B) small dataset, and (C) multi-class dataset. The Y-axis denotes the corrected p-values, and the X-axis represents the individual amino acids. A lower p-value indicates a more significant difference in the frequency of a specific amino acid between THPs and non-THPs.

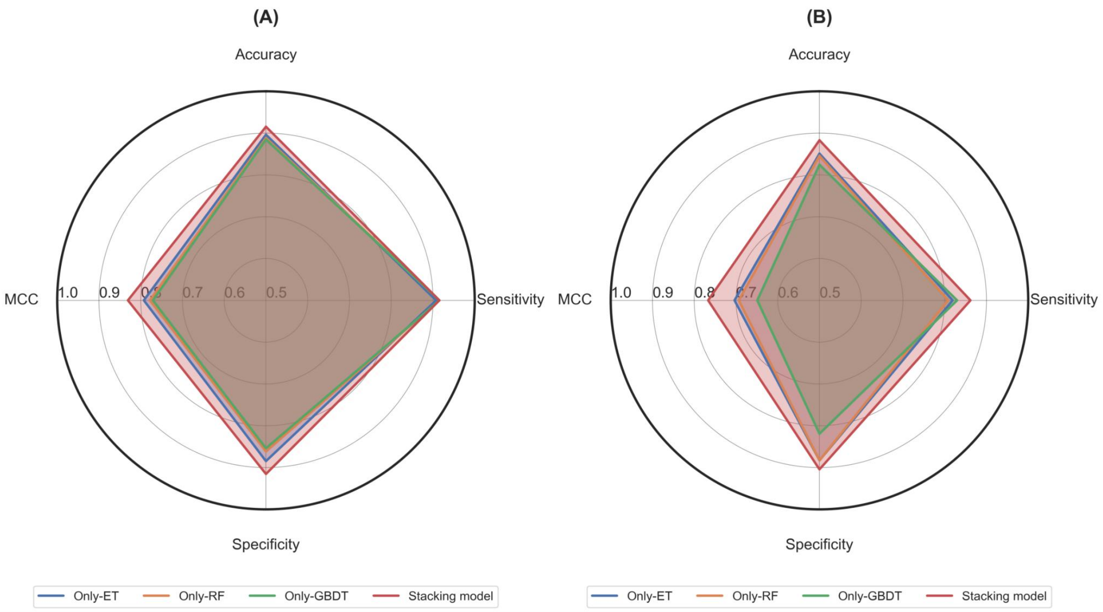

Figure S4. Performance comparison of individual base-learning algorithms and stacking models on two datasets. (A) represent main dataset. (B) represent small dataset.

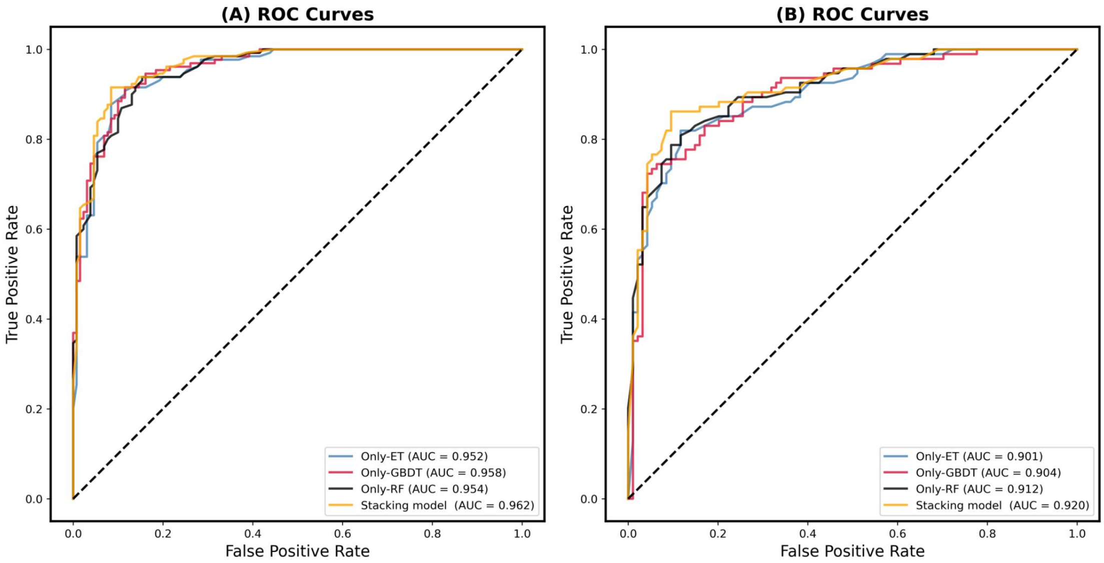

Figure S5. Comparison of the ROC curves of of Individual and Stacking models on two datasets. (A)represents main dataset. (B)represents small dataset.

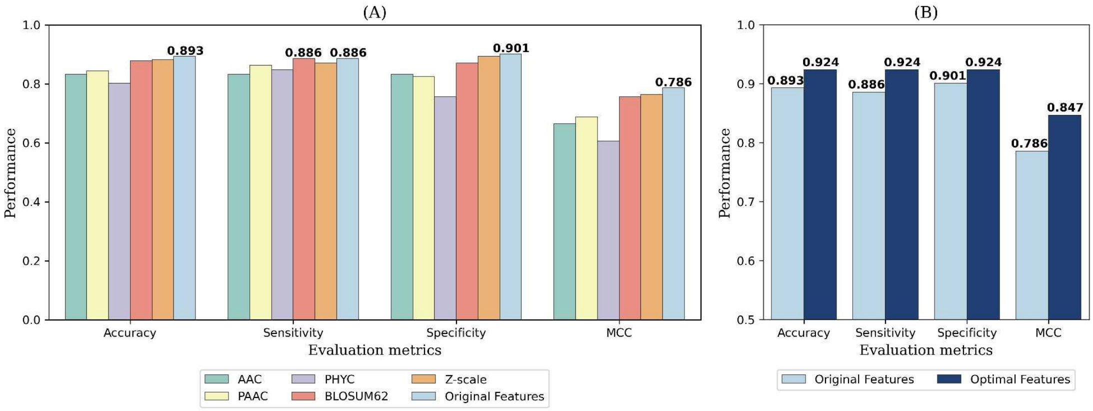

Figure S6. Performance comparison of different features on independent test sets of multi-class independent dataset.

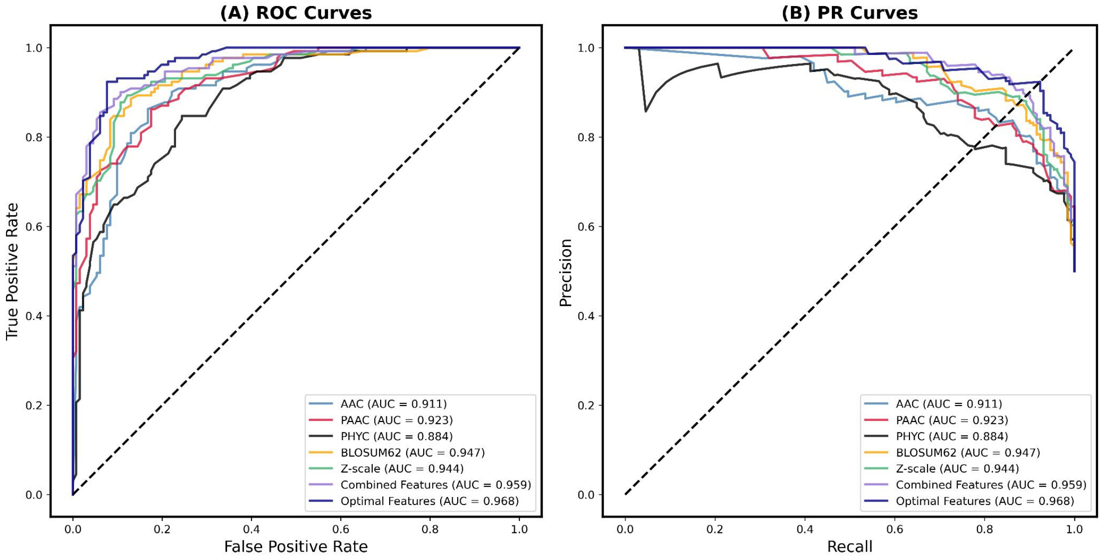

Figure S7. ROC and PR curves of different features on the independent test set of multi-class independent dataset.

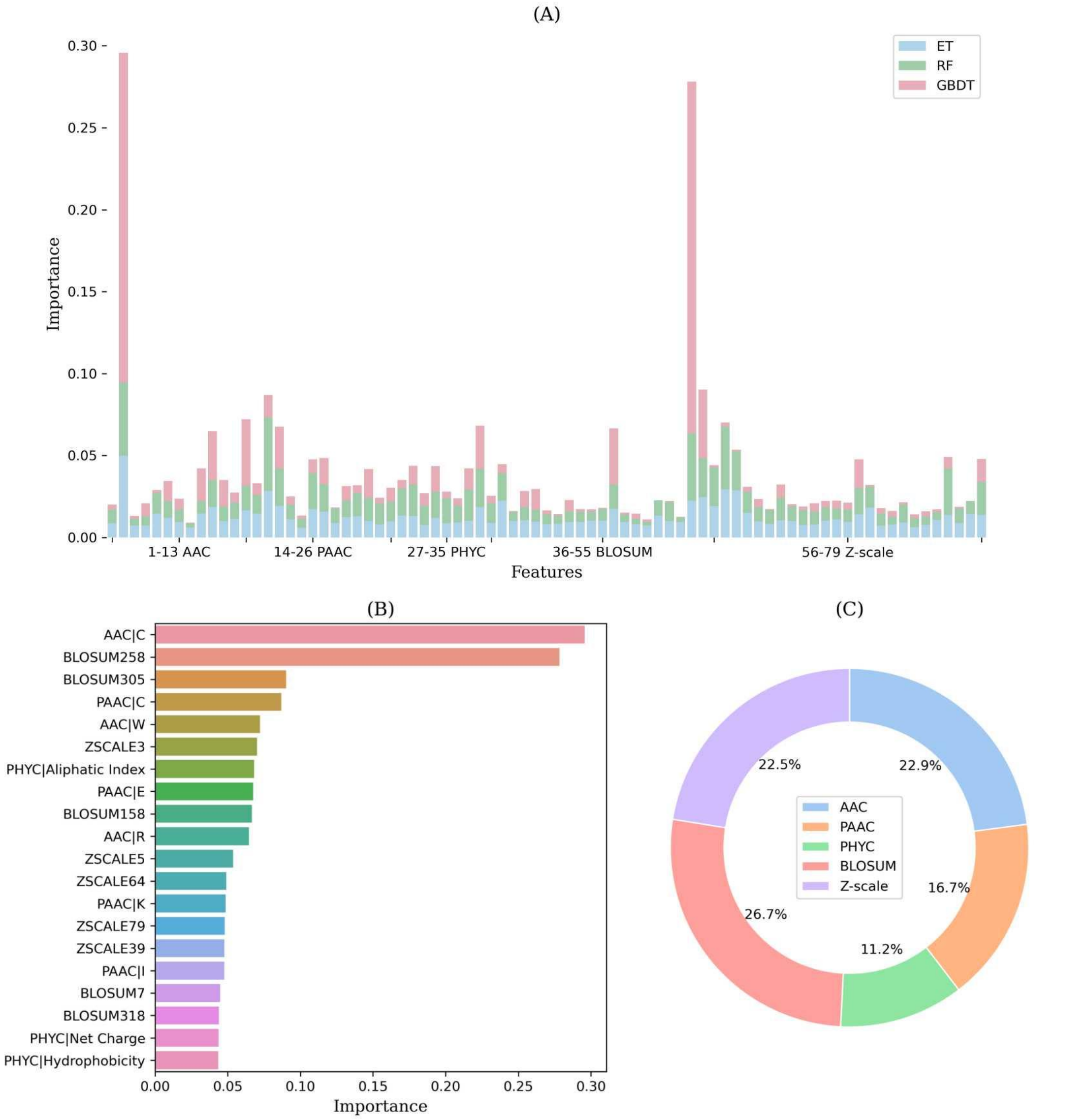

Figure S8. Features importance analysis on main dataset. (A) Visualize the cumulative contribution of each feature, where blue, green and pink represent the feature contribution from ET, RF and GBDT, respectively. (B) Demonstrate the top twenty features with the highest cumulative contribution of each feature. (C) Illustrate the total contribution of each descriptor.

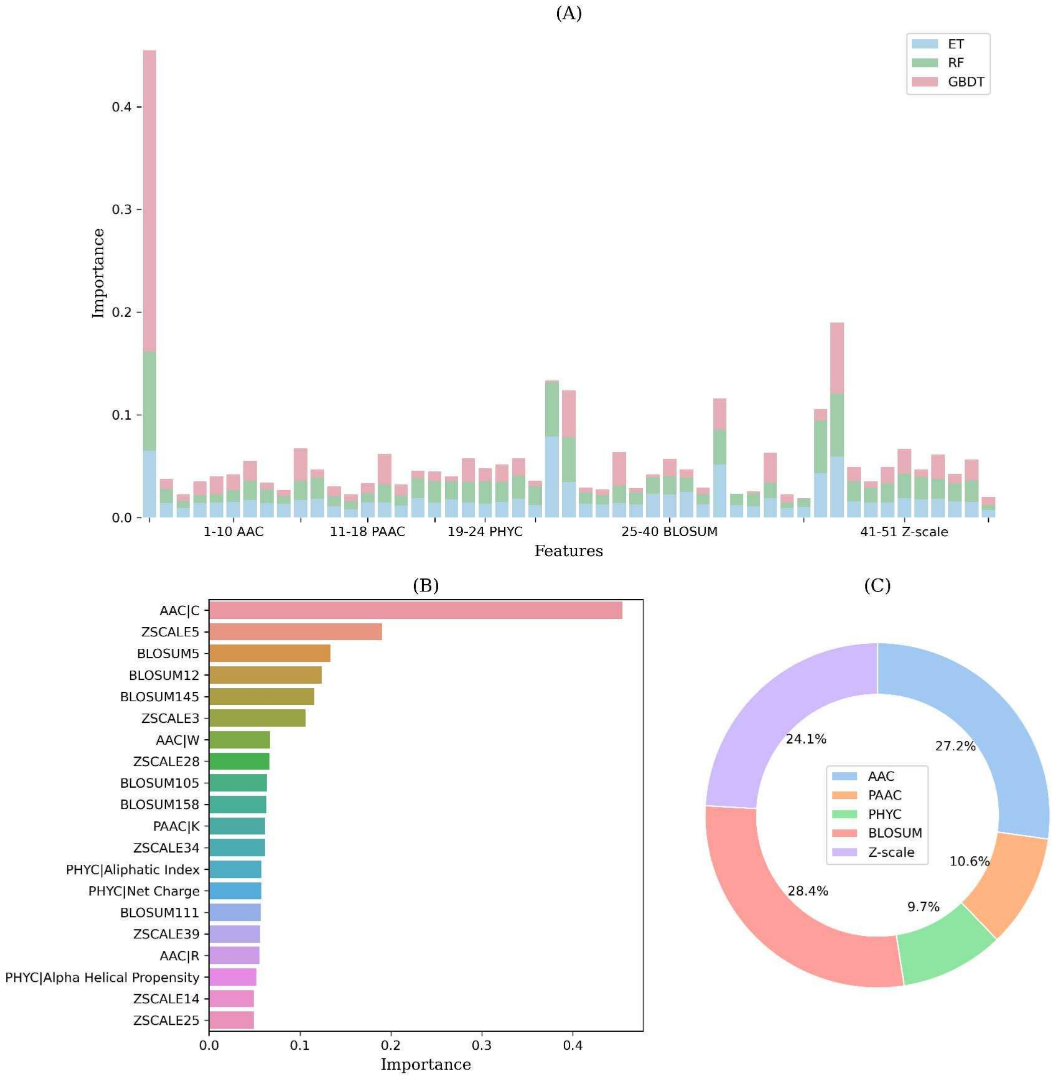

Figure S9. Features importance analysis on small dataset. (A) Visualize the cumulative contribution of each feature, where blue, green and pink represent the feature contribution from ET, RF and GBDT, respectively. (B) Demonstrate the top twenty features with the highest cumulative contribution of each feature. (C) Illustrate the total contribution of each descriptor.

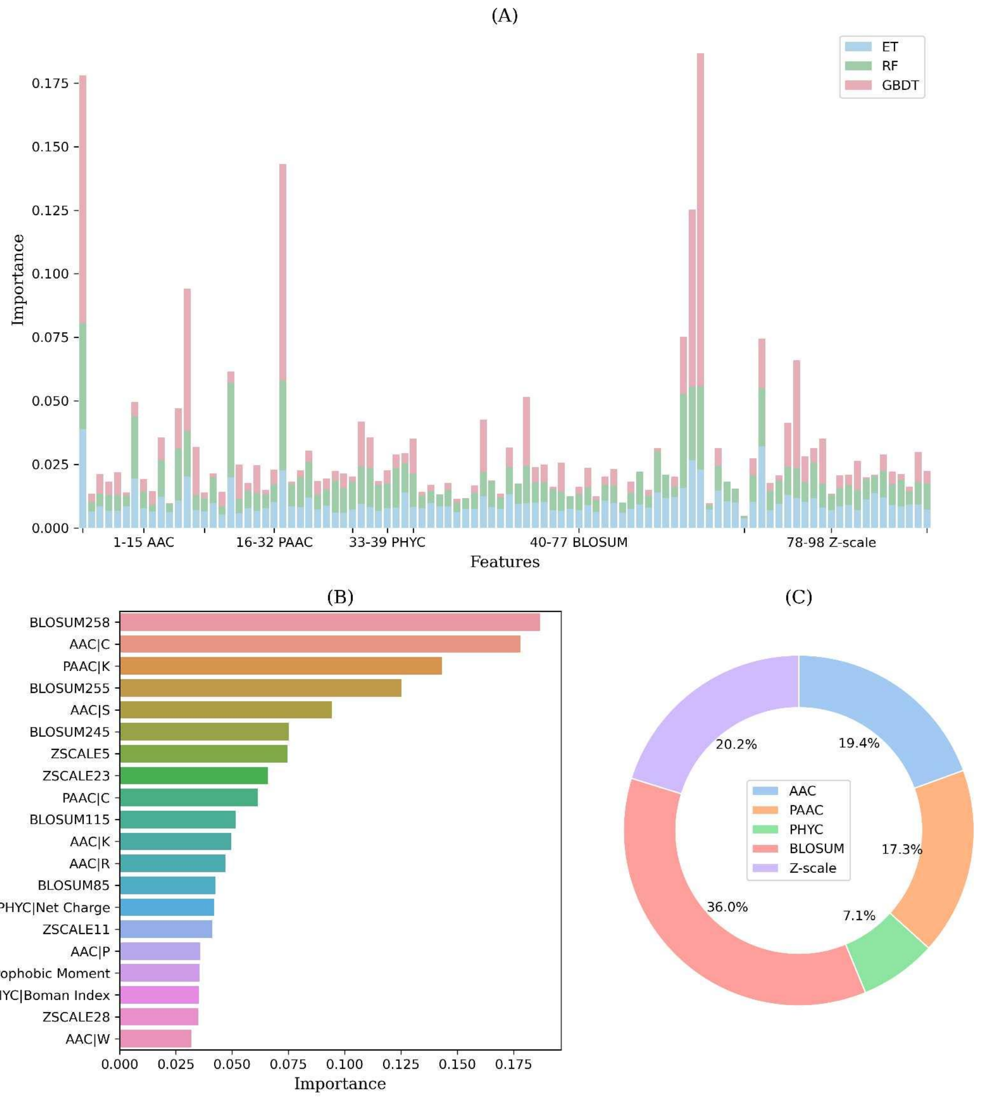

Figure S10. Features importance analysis on multi-class dataset. (A) Visualize the cumulative contribution of each feature, where blue, green and pink represent the feature contribution from ET, RF and GBDT, respectively. (B) Demonstrate the top twenty features with the highest cumulative contribution of each feature. (C) Illustrate the total contribution of each descriptor.
